# Association Between HTLV-1 Genotypes and Risk of HAM/TSP

**DOI:** 10.3389/fmicb.2019.01101

**Published:** 2019-05-15

**Authors:** Mineki Saito

**Affiliations:** Department of Microbiology, Kawasaki Medical School, Kurashiki, Japan

**Keywords:** HTLV-1, HAM/TSP, tax, virus subgroup, susceptibility

## Abstract

Human T-cell leukemia virus type 1 (HTLV-1)-associated myelopathy/tropical spastic paraparesis (HAM/TSP) is a neurological disorder presenting with spastic paraparesis, sphincter dysfunction, and mild sensory disturbance in the lower extremities, which develops in a small minority of HTLV-1-infected individuals. HTLV-1-specific T cells are efficiently activated through dedicated human leukocyte antigen-mediated mechanisms, a process considered deeply involved with its pathogenesis. It has been reported that the lifetime risk of developing HAM/TSP differs between ethnic groups, and there is an association between HTLV-1 *tax* gene subgroups (i.e., *tax* subgroup-A or -B), which correspond to HTLV-1 “cosmopolitan subtype 1a subgroup A (i.e., transcontinental subgroup)” and “cosmopolitan subtype 1a subgroup B (i.e., Japanese subgroup),” respectively, and the risk of HAM/TSP in the Japanese population. These findings suggest that a given host’s susceptibility to HAM/TSP is deeply connected with both differences in genetically determined components of the host immune response and HTLV-1 subgroup. Therefore, it is crucial for ongoing work to focus on developing novel treatments and preventative approaches for HAM/TSP. In this review, based on an overview of the topic and our latest research findings, the role of the HTLV-1 subgroup on the effects of virus–host interactions in the pathogenesis of HAM/TSP is discussed.

## Introduction

Human T-cell leukemia virus type 1 (HTLV-1), the first human retrovirus to be discovered, is etiologically associated with adult T-cell leukemia (ATL) (Poiesz et al., 1980; Hinuma et al., 1981; Yoshida et al., 1984) and HTLV-1-associated myelopathy/tropical spastic paraparesis (HAM/TSP) (Gessain et al., 1985; Osame et al., 1986). In HTLV-1 infection, only a minority of infected individuals develop the disease after prolonged viral persistence (2–5% for ATL and 0.25–3.8% for HAM/TSP), whereas the majority remain asymptomatic carriers (ACs) throughout their lives (Uchiyama, 1997; Bangham and Matsuoka, 2017). However, a definitive answer to the fundamental question of why some HTLV-1-infected individuals develop diseases, whereas the vast majority remain healthy in a lifetime has not yet been answered. Since both ATL and HAM/TSP have unidentifiable causes and/or a lack of curable treatments, evaluation of the individual risk of developing diseases in ACs would certainly be of considerable importance, especially in HTLV-1 endemic areas. This review summarizes past and recent studies of HTLV-1, especially focused on the viral and host genetic risk factors for developing HAM/TSP, in an attempt to answer this fundamental question.

## Host Genetic Factors Governing HAM/TSP Susceptibility

HTLV-1-associated myelopathy/tropical spastic paraparesis is a rare chronic progressive neuroinflammatory disease of the central nervous system mainly involve the thoracic spinal cord, therefore characterized by spastic paraparesis, sphincter dysfunction, and mild sensory disturbance in the lower extremities (Nakagawa et al., 1995). Based on the number of infected individuals, the number of HAM/TSP cases in the world has been estimated to be between 60,000 and 100,000 in HTLV-1 endemic areas. Sporadic cases of HAM/TSP have also been reported from non-endemic areas such as the United States and Europe, among immigrants from endemic areas (Araujo and Silva, 2006). In Japan, by the end of 2018, 551 patients of an estimated 3,000 cases were registered in “HAM-net,” a recently established registration system that collect personal and clinical data from patients with HAM/TSP (Coler-Reilly et al., 2016).

Shortly after the discovery of HAM/TSP, scientists found that *in vitro* culture of peripheral blood mononuclear cells (PBMCs) from HTLV-1-infected individuals exhibit “spontaneous lymphocyte proliferation” (SLP) in the absence of exogenous stimuli such as antigens or mitogens. In patients with HAM/TSP, the levels of SLP reflect the severity of the disease (Itoyama et al., 1988; Ijichi et al., 1989), and the levels of SLP are higher in patients with HAM/TSP than those in patients with ATL or in ACs (Itoyama et al., 1988; Eiraku et al., 1992). Meanwhile, patients with HAM/TSP have significantly higher anti-HTLV-1 titers than those in patients with ATL or in ACs (Nakagawa et al., 1995). These observations raised the possibility that the immune hypersensitivity to HTLV-1 observed in patients with HAM/TSP is governed by host genetic factors. Indeed, just 2 years after the discovery of HAM/TSP, Usuku et al. (1988) identified human leukocyte antigen (HLA)-DR1 and -DR4 as two HLA-DR serotypes highly represented in HAM/TSP, hypothesizing that HAM/TSP and ATL correspond to two populations of HTLV-1 carriers with distinct immune responses to HTLV-1. Later on, they also discovered that large quantities of HTLV-1-specific antibodies are present in the cerebrospinal fluid of patients with certain HAM/TSP-related HLA haplotypes (Kitze et al., 1996) and identified an HLA-DRB1^∗^0101-restricted dominant epitope on HTLV-1 envelope (Env) glycoprotein gp21 (Kitze et al., 1998). They further corroborated the association between host genetic factors and the susceptibility to HAM/TSP by genomic DNA analysis of 224 patients with HAM/TSP and 202 ACs living in Kagoshima Prefecture, located in HTLV-1 endemic southwest Japan (Nagai et al., 1998). Namely, the HTLV-1 proviral load (PVL), which represents the number of HTLV-1-infected cells *in vivo* because HTLV-1-infected cells *in vivo* harbor one copy of the integrated HTLV-1 provirus per cell (Cook et al., 2012), is 7- to 16-fold higher in patients with HAM/TSP than in ACs, and the PVL is significantly higher in ACs with genetic relatives with HAM/TSP than in ACs with no family history (Nagai et al., 1998). Interestingly, they also reported that the risk of HAM/TSP increases exponentially once the HTLV-1 PVL exceeds 1% of PBMCs, providing more evidence to support increased PVL (i.e., number of HTLV-1 infected cells *in vivo*) as a risk factor for HAM/TSP (Nagai et al., 1998). At around the same time, a collaborative study by this Kagoshima group and London group showed that possession of the HLA-class I genes HLA-A^∗^02 and Cw^∗^08 is associated with lower risk of HAM/TSP through a statistically significant reduction in the HTLV-1 PVL, whereas HLA-class I HLA-B^∗^5401 and class II HLA-DRB1^∗^0101 was associated with a higher risk of HAM/TSP in the same population (Jeffery et al., 1999, 2000). Since the protective effect of HLA-A^∗^02 has also been reported in the Brazilian population (Borducchi et al., 2003; Catalan-Soares et al., 2009), HLA-A^∗^02 may play an important role in regulating HTLV-1 infection. Indeed, consistent with these findings, it has been reported that HLA class I-related epitopes targeted by cytotoxic T lymphocytes (CTLs) are most commonly found on HTLV-1 regulatory factor Tax; HLA-A^∗^02-restricted epitope on Tax, spanning amino acids 11–19, is known to provoke a particularly intense immune response (Kannagi et al., 1991, 1992; Parker et al., 1992); HTLV-1 Env gp21 immunodominant epitope is restricted by HLA-DRB1^∗^0101 (Yamano et al., 1997; Kitze et al., 1998); and HLA-DRB1^∗^0101 is associated with the susceptibility to HAM/TSP in independent HTLV-1-infected populations in southwest Japan (Jeffery et al., 1999, 2000) and northeastern Iran (Sabouri et al., 2005). These findings convincingly demonstrated that immune response to HTLV-1 regulated by HLA is an important determinant of the number of HTLV-1 infected cells *in vivo* and the risk of HAM/TSP.

## Ethnic and Geographical Differences in HLA Associations with the Risk of HAM/TSP

As observed in other infections, the reported association between HLA genes and the outcome of HTLV-1 infection shows ethnic and geographical differences. In Iranian HTLV-1-infected individuals, although HLA-DRB1^∗^0101 is associated with susceptibility to HAM/TSP in the absence of HLA-A^∗^02, as observed in Kagoshima, southwest Japan, both HLA-A^∗^02 and HLA-Cw^∗^08 have no effect on either the risk of developing HAM/TSP or HTLV-1 PVL (Sabouri et al., 2005), suggesting that the underlying mechanism involving HLA-DRB1^∗^0101 may not be the same between the Iranian and Japanese populations. Furthermore, although the sample size was small, the lack of association between HLA-A^∗^02 and the risk of HAM/TSP has been reported in other ethnic groups, such as Afro-Caribbean individuals from Martinique (Deschamps et al., 2010), Jamaica (Goedert et al., 2007), and Spain (Trevino et al., 2013). There are several possible explanations for these observed differences as follows: (1) the immunodominant target epitopes recognized by class I major histocompatibility complex (MHC)-restricted CTLs are different among various HTLV-1 strains in different racial and ethnic groups; (2) unknown environmental factors such as co-infection with other pathogens affect HTLV-1 PVL and HAM/TSP risks in different racial and ethnic groups; and (3) different A^∗^02 subgroups in different racial and ethnic groups affect PVL and HAM/TSP risk because HLA-A^∗^02 CTL responders to Tax frequently recognize more than one A^∗^02-restricted Tax epitope (Parker et al., 1994), and A^∗^02 subgroups differ significantly in their peptide-binding characteristics (Barouch et al., 1995; Sudo et al., 1995). Therefore, further studies of ethnic and geographical differences in associations between HLA and outcomes of HTLV-1 infection are needed using sufficient numbers of samples because most HLA alleles occur with relative infrequency and in strong linkage disequilibrium with other alleles. Furthermore, it is also necessary to consider the differences in other genetic factors, including non-HLA genes, and the different types and subtypes of HTLV-1 distributed in diverse racial/ethnic groups.

## Classification of HTLV-1 Subgroups

HTLV-1-infected individuals are widely distributed across different geographical regions, such as sub-Saharan Africa, South Africa, north-eastern Iran, Melanesia (e.g., Solomon Islands, Papua New Guinea), southwest Japan, Australia (i.e., Aboriginals), the Caribbean, and South America (Verdonck et al., 2007; Gessain and Cassar, 2012). In these endemic areas, seven subtypes of HTLV-1 exist (subtypes 1a–1g), defined based on a phylogenetic analysis of viral long terminal repeats (LTRs): 5 African subtypes (subtypes 1b, 1d, 1e, 1f, and 1g), a Melanesian/Australian subtype (subtype 1c), and a cosmopolitan subtype (subtype 1a). Among them, cosmopolitan subtype A has the broadest worldwide distribution of all of them and is further divided into 5 “subgroups” (subgroups A–E of subtype 1a) (Proietti et al., 2005; Wolfe et al., 2005; van Tienen et al., 2012): transcontinental (A), Japanese (B), West African (C), North African (D), and Afro-Peruvian (E) (Gessain and Cassar, 2012). In the Japanese population, the transcontinental (A) and Japanese (B) subgroups predominate, and sequence variation of HTLV-1 *tax* gene determines the “HTLV-1 *tax* subgroups,” i.e., *tax* subgroup-A and subgroup-B correspond to LTR-based “cosmopolitan subtype 1a subgroup A” and “cosmopolitan subtype 1a subgroup B,” respectively (Furukawa et al., 2000). As shown in Table 1, subgroup-specific nucleotide substitutions are observed in viral regulatory genes Tax (on the sense-strand) and HTLV-1 bZIP factor (HBZ; on its anti-sense-strand). Namely, *tax* subgroup-A sequence differed at 4 nucleotides in Tax-coding and 1 nucleotide in HBZ-coding sequences compared with Japanese prototypic ATK-1 strains (Seiki et al., 1983), which result in two amino acid substitutions in Tax and single amino acid substitution in HBZ.

**Table 1:** Nucleotide variations specific to HTLV-1 *tax* subgroup.

	Nucleotide variation and amino acid change in *tax* subgroup^a^				
	HBZ	Tax	Tax	Tax	Tax
	-7247 S→P	7900 NC	7962 A→V	8211 S→N	8347 NC
ATK-1	A	C	C	G	A
*tax* subgroup-B	A	C	C	G	A
*tax* subgroup-A	G	T	T	A	C

^a^*Nucleotide position is provided as aligned with the prototype ATK-1 sequence (GenBank accession number: J02029) (Seiki et al., 1983). Amino acid change results from nucleotide substitutions are shown as single-letter amino acid codes (i.e., tax subgroup-B → tax subgroup-A). NC, no change*.

## Incidence of HAM/TSP in Different Geographical Areas

Although the incidence of HAM/TSP has a two- to three-fold higher risk in women both among Jamaican and Japanese HTLV-1-infected individuals, the annual incidence of HAM/TSP is much higher among Jamaican individuals than among Japanese individuals (20 and 3 cases, respectively, per 100,000). Thus, it is noteworthy that Jamaican individuals have a higher incidence of “cosmopolitan subtype 1a subgroup A” infection (i.e., Jamaican individuals infected with HTLV-1 harboring subgroup-A nucleotide substitutions) compared with Japanese individuals with a higher incidence of the “cosmopolitan subtype 1a subgroup B” infection (Osame et al., 1990; Tajima, 1990; Kramer et al., 1995; Nakagawa et al., 1995; Hisada et al., 2004). Namely, the lifetime risk of HAM/TSP is different among different ethnic groups. The lifetime incidence of HAM/TSP, i.e., the probability that a neonatal carrier of HTLV-1 will develop HAM by 75 years of age, was reported as 0.25% in the Kagoshima prefecture, which is located in HTLV-1-endemic southwest Japan (Kaplan et al., 1990), and this was significantly lower than the lifetime incidence reported in Jamaica and other Caribbean islands (1.9%) (Maloney et al., 1998) and Brazil (9%) (Rosadas et al., 2018). Interestingly, HLA-A^∗^02 provides protection against *tax* subgroup-B, but not against *tax* subgroup-A in both the Japanese (Furukawa et al., 2000) and Iranian (Sabouri et al., 2005) populations. These findings increase the possibility that regional differences in HAM/TSP prevalence can be explained by HLA haplotypes and corresponding geographical variations in HTLV-1 subtypes and subgroups.

Interestingly, HTLV-1 in Iranian individuals, which showed no association between HLA-A^∗^02 and the risk of HAM/TSP, possessed 10 different nucleotides in the *tax* region compared with Japanese prototypic strain ATK-1 (= *tax* subgroup B) (Seiki et al., 1983). Among these, nucleotides 7900, 7962, 8211, and 8347 were identical with “*tax* subgroup A,” whereas there are six more nucleotide substitutions that induced four additional different amino acids in the Japanese *tax* subgroup A (Table 2). Because Iranian *tax* induces HAM/TSP with lower PVL than Japanese strains (Sabouri et al., 2005), it is plausible that the Iranian HTLV-1 strain, which belongs to “cosmopolitan subtype 1a subgroup A,” has a much higher risk for HAM/TSP than the Japanese strains of both *tax* subgroup A and B. Meanwhile, using 211 Japanese samples, Nozuma et al. (2017) recently confirmed previous findings by Furukawa et al. (2000), i.e., transcontinental subgroup of HTLV-1 (= *tax* subgroup A) is susceptible to HAM/TSP compared with the Japanese subgroup (= *tax* subgroup B). These findings suggested that a high-risk subgroup of HTLV-1 explains the observed differences in HTLV-1 PVL and higher risk for HAM/TSP.

**Table 2:** Nucleotide variations specific to Iranian HTLV-1 Tax.

	Nucleotide variation, by position and amino acid change^a^									
Subgroup (n)	7625 M→V	7814 I→V	7858 NC	7900 NC	7962 A→V	7994 N→H	8211 S→N	8316 G→E	8317 NC	8347 NC
ATK-1 (= *tax* subgroup-B)	A	A	T	C	C	A	G	G	C	A
*tax* subgroup-A	A	A	T	T	T	A	A	G	C	C
Iranian *tax*	G	G	C	T	T	C	A	A	G	C

^a^*Nucleotide position corresponds to that of prototypic strain ATK-1. Amino acid change results from nucleotide substitution. NC, no change*.

## Nucleotide Sequence Variation of HTLV-1 in Patients With HAM/TSP

Since HTLV-1 infection precedes both HAM/TSP and ATL, the possibility that certain HTLV-1 strains cause one condition and others cause the other has been investigated. However, the resounding conclusion was that no such disease-specific strains existed (Daenke et al., 1990; Kinoshita et al., 1991). Nonetheless, later work identified mutations in the region of the viral genome coding for *tax* associated with HAM/TSP morbidity (Kira et al., 1994; Renjifo et al., 1995). In one of these studies, mutant proviral *tax* sequences were frequently isolated from lesioned tissue sampled from the spines of patients with HAM/TSP (Kira et al., 1994). Subsequently, proviral sequences with similar mutations were found in PBMCs from ACs, proving both that *tax* mutants are not unique to patients with HAM/TSP and that infected cells do not exclusively accumulate around spinal cord lesions (Saito et al., 1995). Another study found a single point mutation, located at nucleotide 7959 of the *tax* region, to be associated with HAM/TSP status (Renjifo et al., 1995). However, later work showed it to be specific to virus strains of a specific geographical area, rather than a disease-specific mutation (Mahieux et al., 1995). These findings led many to conclude that there were simply no HAM/TSP-specific mutations in the HTLV-1 genome.

**Figure 1:**
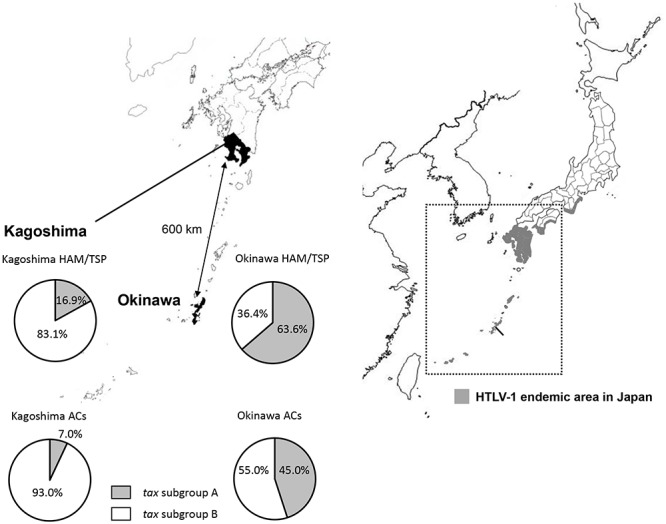
Geographical distribution of HTLV-1 *tax* subgroups in Kagoshima and Okinawa Prefecture, Japan. The distribution of HTLV-1 *tax* subgroups varied by geographic region in southern Japan. A significantly higher prevalence of *tax* subgroup-A was found in Okinawa prefecture than Kagoshima prefecture both in HAM/TSP patients and asymptomatic carriers (ACs) (Okinawa vs. Kagoshima, HAM/TSP: 63.6 vs. 16.9%, *p* < 0.001, ACs: 45.0 vs. 7.0%, *p* < 0.001, *p*-value assessed by χ^2^ test).

Then, in Furukawa et al. (2000) reported the existence of two *tax* subgroups, i.e., “*tax* subgroup-A” and “*tax* subgroup-B,” with distinct sequences based on an analysis of a large number of specimens from patients with HAM/TSP and ATL and from ACs in Kagoshima Prefecture, located in HTLV-1 endemic southwest Japan. Most interestingly, they found *tax* subgroup-A carriers to be at about 2.5 times the risk of developing HAM/TSP compared to *tax* subgroup-B carriers, independent of HLA haplotype (Furukawa et al., 2000). As already mentioned, they explicitly showed that *tax* subgroup-A and -B correspond to the transcontinental (A) and Japanese (B) subgroups within cosmopolitan subtype 1a, a taxonomy defined based on LTR sequence similarity (Furukawa et al., 2000). Table 3 and Figure 1 show the results of our analysis of the *tax* subgroups of patients with HAM/TSP and ACs living in Kagoshima and Okinawa Prefectures. Okinawa prefecture consists of 160 islands, of which 49 islands are inhabited, and is located in the subtropical southern-most point of Japan. In Okinawa prefecture, *tax* subgroup-A and -B, respectively, comprised 63.6 and 36.4% of patients with HAM/TSP: moreover, *tax* subgroup-A was significantly more common among ACs here than in Kagoshima. Kagoshima had significantly more *tax* subgroup-B carriers, but the HAM risk was higher among *tax* subgroup-A carriers; curiously, however, the *tax* subgroup was not associated with any differences in HAM/TSP susceptibility in Okinawa, despite the higher frequency of *tax* subgroup-A (Table 3). In future studies, it is important to examine the HAM/TSP prevalence rates in these two prefectures to clarify whether or not the pathogenesis of HAM/TSP is influenced at all by the HTLV-1 subgroup.

**Table 3:** HTLV-1 tax subgroup-A is associated with the risk of HAM/TSP in Kagoshima but not in Okinawa, Japan.

Population	HAM/TSP		ACs					
	*tax* subgroup-A	*tax* subgroup-B	*tax* subgroup-A	*tax* subgroup-B	χ^2a^	P	OR	CI, 95%
Kagoshima	41 (16.9%)	201 (83.1%)	14 (7.0%)	186 (93.0%)	9.04	**0.0264**	2.71	1.43–5.13
Okinawa	21 (63.6%)	12 (36.4%)	9 (45.0%)	11 (55.0%)	1.08	0.298	1.76	0.69–6.63

^a^*Reported as one-tailed with Yates correction. Odds ratio used the approximation of Woolf. The significant value is indicated in bold. OR, Odds ratio; CI, confidence interval; HAM/TSP, HTLV-1-associated myelopathy/tropical spastic paraparesis; ACs, asymptomatic carriers*.

## Tax Subtype-Specific Functional Differences

We have previously reported that although no clear differences were noted in clinical and laboratory characteristics, clinical course, and treatment response between HAM/TSP patients with subgroup-A and subgroup-B, different HTLV-1 subgroups are characterized by different patterns of viral and host gene expression in patients with HAM/TSP (Yasuma et al., 2016). Namely, *HBZ* mRNA expression was significantly higher in patients with HAM/TSP with *tax* subgroup-B than in those with *tax* subgroup-A in HTLV-1-infected cells, and there is a positive correlation between the expression of *HBZ* mRNA and its target *Foxp3* mRNA in patients with HAM/TSP with *tax* subgroup-B, but not in patients with *tax* subgroup-A. It has previously been reported that Tax suppresses (Arnulf et al., 2002; Lee et al., 2002) and HBZ enhances (Zhao et al., 2011) transforming growth factor (TGF)-β-mediated signaling activity and HBZ induces *Foxp3* expression in naïve T cells via Smad3-dependent TGF-β signaling (Zhao et al., 2011). We therefore examined whether there were any differences in the ability of subgroup-specific Tax or HBZ to activate the *Foxp3* promoter. However, no functional differences were observed between the Tax and HBZ proteins of each subgroup (i.e., HBZ-A and HBZ-B or Tax-A and Tax-B) based on reporter gene assays using three different reporters, i.e., the HTLV-1-3′-LTR-promoter luciferase reporter, human Foxp3 promoter luciferase reporter, and human TGF-β-responsive concatemer-containing luciferase reporter (Yasuma et al., 2016). Meanwhile, co-expression of Tax did not significantly influence the function of HBZ to activate the Foxp3-promoter in transfected cells (Yasuma et al., 2016). These results indicate that different patterns of viral and host gene expression in patients with HAM/TSP observed in different HTLV-1 *tax* subgroups are characterized via independent mechanisms of direct transcriptional regulation.

More recently, we further studied the function of each subgroup-specific Tax using microarray analysis, reporter gene assays, and evaluation of viral–host protein–protein interactions (Naito et al., 2018). In this study, we transformed human acute T-cell leukemia cell line Jurkat Tet-On cells with a construct containing either full-length *tax* subgroup-A or -B gene downstream of a tetracycline-responsive promoter, and then used comprehensive microarray analysis to identify genes of which expression levels changed following Tax protein induction. Interestingly, induced Tax protein (i.e., Tax-A or Tax-B) potently activated the expression of their target genes, and Tax-A and -B elicited the expression of different combinations of both already-reported and unreported target genes (Naito et al., 2018). Furthermore, although the chemokine CXCL10, which has been proposed as a prognostic biomarker for HAM/TSP (Sato et al., 2013), was more efficiently induced by Tax-A than by Tax-B through the nuclear factor (NF)-κB pathway, there was no difference in the ability of each subgroup of Tax (i.e., Tax-A or Tax-B) to activate the CXCL10 gene promoter evaluated using a reporter gene assay with HTLV-1-negative human T-cell line Jurkat (Naito et al., 2018). Meanwhile, chromatin immunoprecipitation assays revealed that the ternary complex containing Tax-A is more efficiently recruited onto the CXCL10 promoter, which contains two NF-κB binding sites, than that containing Tax-B (Naito et al., 2018).

## Concluding Remarks

As discussed above, each subgroup-specific Tax or HBZ have a unique expression signature of both viral and host genes, suggesting that differential expression of pathogenesis-related genes by subgroup-specific viral regulatory proteins may be associated with the onset of HAM/TSP. However, the associations between HAM/TSP susceptibility and *tax* subgroups still remain to be fully clarified. Moreover, to date, no research has observed subgroup-based differences in the ability of Tax to regulate transcription via independent mechanisms of direct transcriptional regulation nor compared HTLV-1 immunogenicity between subgroups A and B. Future research in these directions is eagerly anticipated to identify attractive targets for novel therapeutics for HAM/TSP.

## Author Contributions

MS wrote this review manuscript.

## Conflict of Interest Statement

The author declares that the research was conducted in the absence of any commercial or financial relationships that could be construed as a potential conflict of interest.
